# Multi-Domain Indoor Dataset for Visual Place Recognition and Anomaly Detection by Mobile Robots

**DOI:** 10.1038/s41597-025-05124-3

**Published:** 2025-05-19

**Authors:** Piotr Wozniak, Tomasz Krzeszowski, Bogdan Kwolek

**Affiliations:** 1 Faculty of Electrical and Computer Engineering, Rzeszow University of Technology, Rzeszow, 35-959 Poland; 2 Faculty of Computer Science, AGH University of Krakow, Krakow, 30-059 Poland

**Keywords:** Computer science, Scientific data

## Abstract

Visual location recognition encompasses place recognition (PR) and anomaly detection (AD). These are crucial tasks for autonomous robots to accurately determine the location and the occupied place. To accelerate research in this area, we introduce a multi-domain dataset for indoor visual place recognition and anomaly detection by mobile robots. The dataset includes 89,550 RGB images captured in nine rooms. The data collection process involved both manual recordings and recordings captured by mobile robots. The images depict a wide range of scenarios, including variations in lighting, robot vision, and human activity. Additionally, we provide an analysis of other available datasets referenced in the literature. This article presents a freely available dataset for research on place recognition and presents an example application in the field of anomaly detection. The baseline methods were thoroughly tested and achieved an 80.18% accuracy in anomaly detection for single images and 80.63%-84.18% for image sequences. The article includes a comprehensive presentation of the characteristics of individual image sequences and the most significant conclusions drawn from the research.

## Background & Summary

The deployment of mobile robots for various tasks requires the development of methods that can adapt to environmental changes and modifications. The layout of indoor environments evolves over time, potentially impacting the autonomous operation of robots. As anomalies and modifications occur, it becomes necessary to update the perception systems in use. These changes can significantly disrupt autonomous navigation^1–3^, location detection^4^, place recognition^5–8^, visual place recognition (VPR)^9–11^, SLAM (Simultaneous Localization and Mapping)^12^, and visual loop closure detection^13^. Furthermore, anomalies can pose serious risks and lead to robot failures or slowdowns. To enable unsupervised operation of any robot type, it is essential to rigorously assess and ensure the robustness of its systems in handling anomalies. Comprehensive datasets and evaluation scenarios play a crucial role in the development of autonomous systems. However, collecting the appropriate data and identifying anomalies can be challenging. Anomalies can be related to the environment, the robot itself, or its sensors. Research to date has considered the benefits and drawbacks of proximity sensors^14^, LiDARs^15^, and depth-sensing cameras^16^ in mobile robotic applications. An alternative approach involves utilizing low-cost RGB cameras, which lack depth information^17^. In all the mentioned scenarios, various anomalies arise^18^. When analyzing visual information, it is important to consider not only depth inconsistencies^19^ but also variations in lighting conditions^20^. Given the broad spectrum of potential applications, this article focuses on utilizing RGB cameras integrated into mobile robots. In many robotic tasks, the information captured in the image is sufficient to perform appropriate actions. Objectives such as patrolling^21^ and scene recognition^22^ can be effectively addressed using visual methods, although complex tasks may benefit from additional sensors.

In the literature, the robotics research community often prioritizes cameras and computer vision over a broader range of robot sensors. In computer vision, many modern methods based on machine learning and deep learning have emerged to cope well with tasks such as localization^23^ and navigation^24^. Using a convolutional neural network (CNN)^25^ in various variants allowed everyone to reduce the limitations of approaches based on hand-crafted descriptors. Methods that determine hand-crafted local features such as SIFT^26^ and SURF^27^ or global features such as HOG^28^ are now frequently replaced by learned features^29^. This allowed for better results on noisy data. Recent studies have demonstrated that the integration of advanced deep neural networks and machine learning techniques enables robots to enhance perception, to adapt to complex environments, and improve overall task performance. This work also focuses on the use of learned methods for the problem of scene analysis and anomaly detection. It was assumed that robots have access to sufficient resources in the form of memory and computing capabilities. There are no limits in which we want to execute deep neural networks such as AlexNet^30^, ResNet-50^31^, VGG16^32^. The integration of onboard computing units, along with advanced APIs and learning frameworks, enables the training and fine-tuning^33^ of models, allowing robots to adapt to changing environments and improve their performance in complex tasks. Modern autonomous robots with on-board GPU support running more complicated models and applications^34^. Effective methods were proposed for problems such as SLAM^35^ or camera position estimation^36^. Intelligent systems designed to solve the above problems also extend their operation by trying to measure their own confidence levels and increase knowledge to ultimately achieve better performance. These processes involve detecting and recognizing difficult situations in which decision confidence may be low^37,38^. This may occur when there are too many anomalies or when the method extends outside the intended workspace.

For proper robot’s decision-making when performing a task in unknown conditions, the ability to detect unusual situations is essential. Therefore, the issue of anomaly detection^39^ is an important problem in robotics and beyond. Outlier detection is a fundamental component of many systems. This task can also be treated as a goal in itself. Examples include detecting undesirable activities in information systems^40,41^, visual detection of faults in production^42^, and video analysis to detect threats in monitoring^43^. Similarly, in robotics, anomalies can be treated as hardware failures, unknown rooms^44^, and objects that should not be present in the environment^45,46^. Detecting the above-mentioned anomalies allows the robot to operate autonomously. Previous image-based work focused on visual anomaly detection^9,47,48^. Anomaly detection can be divided into: unsupervised, semi-supervised, and supervised. The detection of anomalies can be approached at the image level^47^ and pixels level^49^. Both approaches are used for different problems and implemented with different approaches. In our work, we rely on the whole images, and the preferred approaches are density estimation^50^, one-class classification^51^, image reconstruction^52^, and self-supervised classification^53^. Due to the challenge of detecting unknown rooms, special attention was given to the Autoencoder-based approach^39,54,55^, and One-Class Support Vector Machine (OCSVM)^56–58^. Based on a review of the literature, Talagala *et al*.^59^ assessed the usefulness of the mentioned methods in the discussed research area.

Before collecting new data, the available datasets were analyzed for problems such as identifying locations with various anomalies. The review referred to sets for robots working in indoor rooms. Attention was also paid to datasets that have a significant impact on the visual recognition of the place occupied by the robot. The articles concerned categorization, navigation, and recognition of places with anomalies. There are various types of disruption, such as changes in lighting, motion blur^60^, human activity, or replacement of the operated robot. It is important that the data reflect the conditions of places where an autonomous robot may be located. There are several techniques that discuss these types of challenges in the literature. Each method approaches the topic of anomalies in a different way. The focus was on sets that presented or simulated the robot’s movement indoors. Important information was how the camera was operated and whether it was placed on the robot. By changing the location of the camera on the robot, the machine’s perception of the scene is different. The selected works proposed sets in which, with the help of a moving human or a tripod^61^, they tried to get close to the effect obtained with an autonomous robot. These approaches enable the recording of images from many places where the robot encounters navigation difficulties, e.g., stairs, elevators, narrow spaces, and door sills. Another important feature of the collections was their structured split. Different approaches are used depending on the rooms and robots that are recorded. Some collections focus on capturing more images from unrelated places of the same type. The idea is to try to classify specific rooms^62^ or types^63–65^ of more general use, for example, bedrooms, bathrooms, and corridors. When the robot navigates, more sequences are collected under different conditions in selected rooms. The difficulty of such sets are anomalies at the level of different lighting conditions or equipment^5,23,66,67^. For example, the dataset^67^ introduces a variety of object types and environmental modifications, aiming to diversify the conditions under which the vision system operates.

The result of the review is a Table 1 presenting datasets available in the literature that match the scope of this work. The table lists datasets used in works related to the topic of this paper. The columns provide specific literature items, information about the recording method and environment, the number of images, and the features of the data collection. Information about the occurrence of anomalies and the characteristics of the set is placed in the column called ’Covariate factors’. We present the number of sequences, the occurrence of anomalies in the sequences, the sequential nature of the images, the utilization of multiple robots, and the presence of individuals in the scene. Most of the datasets presented in Table 1 contain only some of the mentioned features. Hence, the proposed dataset contains all the covariate factors identified in the review of the literature. Additionally, a new feature introduced is large view-point change for various cameras, those mounted on robots. This allows for a broader visual analysis of recorded locations and tests for various robots working under changing conditions. The MDDRobots dataset stands out by offering a broader range of covariate factors, including environment anomalies, sequential images, various robots, human activity, and different cameras. While most datasets focus on limited aspects, MDDRobots integrates multi-aspect variations. It also includes recordings from both manual and robot-based acquisitions, enhancing diversity and robustness. The involvement of a humanoid robot underlines the importance of place recognition for safe and adaptive locomotion, especially due to the need to adjust movement to varying floor conditions. This dataset fills the gap in existing resources by offering a realistic, diverse, and comprehensive collection that more accurately represents real-world conditions for visual place recognition and anomaly detection.

**Table 1:** Comparison of the major indoor place recognition databases for mobile robots.

Database	Mounted Camera	Subsets	Number of Sequences	Rooms	Images	Views	Environment	Covariate Factors	Year
The KTH-IDOL2 Database^5^	robot	2	24	5	22,803	front	indoor	environment anomalies, sequential images, different robots, human activity	2007
COsy Localization Database^66^	robot	3	76	33	140,000+	front, omnidirectional	indoor	environment anomalies, sequential images, different robots, human activity	2009
Indoor67 Dataset^64^	human	1	67	11	15,620	front	indoor, outdoor	non-sequential images	2009
Visual Place Categorization Database^63^	human	6	13	—	51,337	front	indoor	sequential images	2009
York University 11-places Dataset^23^	robot	3	12	17	13,752	front	indoor	environment anomalies, sequential images, different robots	2016
Places365 Dataset^65^	human	1	434	—	2,168,460	front	indoor, outdoor	environment anomalies	2017
Robot@Home Dataset^91^	robot	1	81	36	87,000+	front	indoor	environment anomalies, sequential images	2017
Wozniak *et al*.^62^	robot	1	11	11	8,000	front	indoor	sequential images	2018
Similarly, Chen *et al*.^61^	human	3	69	69	6,800+	front	indoor	sequential images	2018
Hazards&Robots: A Dataset for Visual Anomaly Detection in Robotics^67,92^	robot	3	20	3	324,408	front	indoor	environment anomalies, sequential images, human activity	2023
MDDRobots (proposed)	robot, human	5	171	9	87,750	front	indoor	environment anomalies, sequential images, different robots, human activity, different cameras	2025

The main contribution of this paper is the dataset for visual place recognition and anomaly detection by mobile robots. The prepared set focuses on a multi-domain approach and multi-aspect anomalies occurring in indoor environments. In the context of the VPR problem for mobile robots, we define the multi-domain approach as encompassing various cameras, different robot platforms, diverse lighting conditions, various place layouts, varying presence of people, and different types of robot motion. As an example of the application of the set, the results of the detection of visual anomalies^47^, such as unknown locations, are presented. The use of learned methods for place recognition can also be applied to the task where the recognized place is unknown. Based on the conducted literature review, the following gaps can be identified in the available data collections:

- lack of datasets containing both recordings collected by humans and various types of robots;
- unavailability of multi-domain robot recordings with multiple cameras;
- gap in place recognition tests for multifaceted anomalies by robots.

Given the limitations of current datasets and existing approaches, the key contributions of this article are outlined as follows:

- review and analysis of literature and datasets discussing anomaly detection in VPR;
- a Multi-Domain Dataset for Robots (MDDRobots) with different anomalies collected by multiple robots and humans;
- experimental results on visual detection of anomalies in the form of unknown rooms;
- MATLAB scripts of example methods for the detection of unknown places.

The motivation behind this work is to address the need for accurate visual place recognition and anomaly detection by autonomous robots in indoor environments. Existing datasets are often limited in diversity and do not adequately capture multi-aspect variations, including different robots, environmental conditions, and cameras.

## Methods

**Fig. 1:**
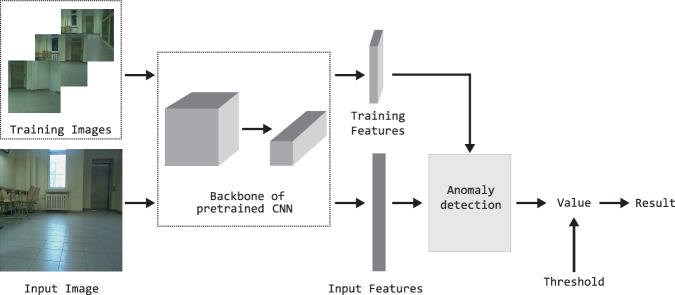
Scheme for CNN-based anomaly detection.

**Fig. 2:**
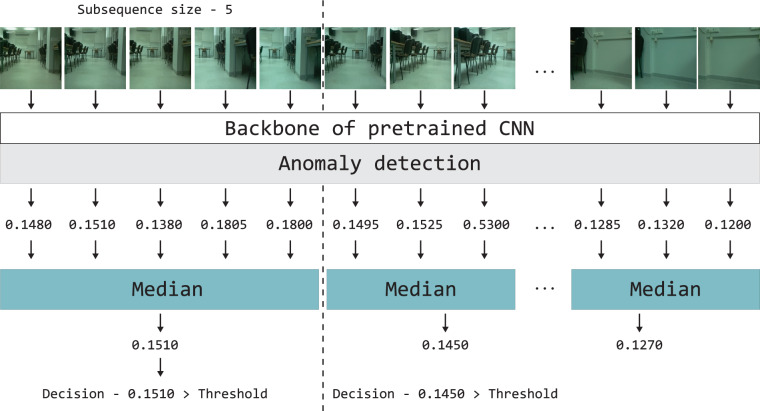
Scheme of an anomaly detection method for image sequences.

The article focuses on the detection of anomalies at the image level. Thus, it is necessary to obtain the final result for the scene view, even when performing a pixel-level analysis. A semi-supervised approach is employed to address the problem of recognition of unknown places. The method relies on information from known rooms, as all data from unknown rooms are considered as anomalies and cannot be accessed during a preprocessing stage. Figure 1 illustrates the general anomaly detection scheme for an unknown location. The anomaly detection method is adapted to images captured within the recorded room. The input images were acquired through manual recordings and recordings by mobile robots equipped with low-cost RGB cameras, capturing 89,550 images from nine rooms over an extended period. Various angles, positions, and conditions, including changes in lighting, cameras placement, and human activity, were considered to ensure the dataset’s robustness for VPR and AD. The features are extracted by the CNN and utilized for training the detection model. During the training stage, the parameters (min and max values) for future normalization to the range from 0 to 1 are defined. These parameters were used during the evaluation of the method. The method analyzes acquired input features to determine if the sample is normal or an outlier. At the image level, a numerical representation of the result is determined, with the specific value depending on the detection method used. The final decision regarding an individual image depends on the output of the method and the threshold set for each method individually. The outcome of the method is influenced by the accuracy of the image description, the method used, and the correctness of the selected threshold value. A possible modification to the anomaly detection method is the use of image sequences. Existing literature presents algorithms that extract insights from data sequences^68,69^. A less demanding option is to aggregate results from individual frames. This approach does not require data preparation for different sequence variants. The method uses a median^70^, which limits the impact of falsely alarming outliers in the analyzed image sequence. This is a common situation for a human or robot during recording. Figure 2 is an extension of the previously given scheme. The images are processed by the CNN network and the anomaly detection method.

### Features extraction

**Fig. 3:**
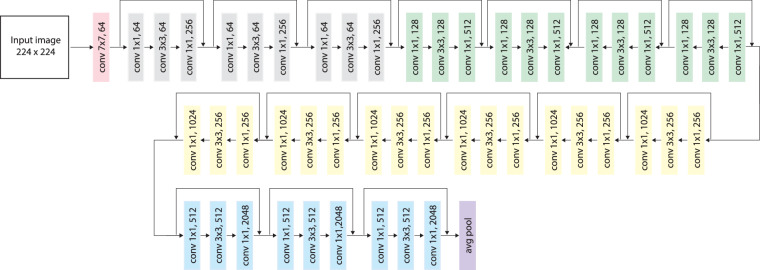
ResNet-50 backbone structure diagram.

The anomaly detection methods are based on an identical approach to obtain a description of images. Features representing rooms were extracted by the pretrained ResNet-50^31^ network using the approach presented in the article^71^. The convolutional neural network determines a feature vector at the end of its backbone (after feature pooling). The final image representation has 2048 features. Figure 3 shows the structure of the ResNet-50 network. The RGB image was fed to the network after scaling to a size of 224 × 224 px. The utilized network was not subject to any modifications or weight changes. The default CNN weights obtained by training on the ImageNet^72^ dataset were used. The features are extracted by the pretrained backbone of the ResNet-50 deep neural network.

### Anomaly detection methods

The following section presents selected anomaly detection methods for the entire image. The decision to choose certain algorithms was conditioned by a review of the literature and the potential implementation of the method on the mobile robot. It was important that the method handled multidimensional data extracted from a convolutional neural network. Isolation Forest (IF)^73–75^, One-Class Support Vector Machine^76,77^, and Autoencoder (AE)^78^ are selected as base methods for further comparisons.

#### Isolation Forest

Isolation Forest^73–75^ is based on a binary tree structure. The method assumes that normal samples are located considerably from the main node. This is in contrast to outlier samples, which are sensitive to the segmentation and are closer to the main node of the tree. The relevant literature^73^ states that IF has the ability to detect anomalies represented by high-dimensional features. This allows it to be selected as an adequate method to support CNN feature vectors. However, there are sets in which deterioration of results was observed due to high dimensionality^79^. There are examples of modifications to the IF-based approach that refer to deep learning. Deep Isolation Forest (DIF)^80^ was introduced, which enables non-linear partitioning on subspaces of varying sizes. This eliminates difficulties in complex datasets with high-dimensional/non-linearly separable data spaces. Similarly, in the case of image analysis, IF acts as a function to verify the allocation of feature representations of normal or outlier data^81^.

#### One-Class Support Vector Machine

One-class classification aims at identifying objects (images) of a specific class among all classes. A variant of this approach is the One-Class Support Vector Machine^82^, which attempts to construct decision boundaries for the normal class in the feature space. A closely related method is Support Vector Data Description (SVDD)^83^, designed to enclose normal data within a hypersphere, offering an alternative formulation for anomaly detection. The method is useful for small amounts of training samples. However, it may be susceptible to the problem with higher-dimensional data. Therefore, there may be a problem when the image representation consists of too many features. The feature representation was the result of CNN, but the final decision was made based on boundary evaluation and a predefined threshold. In the literature, some approaches use a mixture of a single-class classifier and a deep convolution network^56^. Using transfer learning, it is possible to adapt the model to a new problem and then use a one-class classifier. An important extension is the detection of anomalies for multiple classes, rather than a single one^84^. There are also known end-to-end solutions in the form of a one-class classification neural network (OCCNN)^85^. The approach discussed in this paper combines OCSVM and deep learning.

#### Autoencoder

**Fig. 4:**
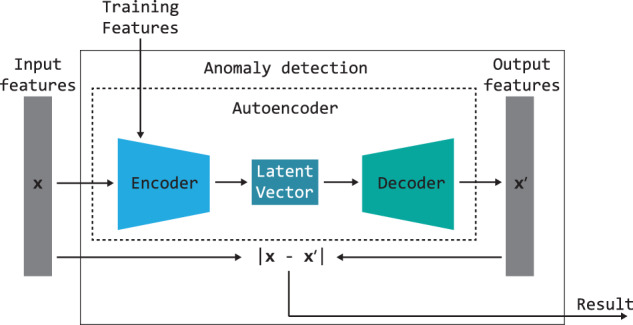
Diagram of the method scheme for anomaly detection based on Autoencoder.

The idea behind Autoencoder is to calculate the data embedding and reconstruct the input^86^. The AE Encoder calculates a low-dimensional data representation in the form of a vector (Latent Vector). Then reverse data reconstruction is performed. There are many approaches and applications of the Autoencoder available in the literature^87,88^. The method can refer to various data, such as feature vectors or images. The approach used in this work is based on an Autoencoder into which features from the CNN network are fed. This reduces the need to train the entire model. An Autoencoder is made of an Encoder and a Decoder. Figure 4 shows the components of the proposed method, corresponding to the Anomaly Detection block presented in Figures 1 and 2. An Encoder has been trained to compress input data by removing redundant or irrelevant information. For the anomaly detection problem, the original feature vector (${\bf{x}}$ - input features) and that after reconstruction (${\bf{x}}{\rm{{\prime} }}$ - output features) are compared. The distance between the vectors was obtained using the cosine distance^89^ and is treated as the result of the AD block. The distance is calculated on the basis of the following equation:

1 $$\cos \theta =\frac{{\bf{a}}\cdot {\bf{b}}}{| | {\bf{a}}| | \cdot | | {\bf{b}}| | }=\frac{{\sum }_{i=1}^{n}{a}_{i}\ast {b}_{i}}{\sqrt{{\sum }_{i=1}^{n}{({a}_{i})}^{2}}\ast \sqrt{{\sum }_{i=1}^{n}{({b}_{i})}^{2}}}$$

where **a** denotes the input features vector, **b** stands for the output features vector, whereas *n* is the length of vectors **a** and **b**.

For the input image, it is necessary to obtain a representation in the form of a feature vector. The features are obtained from the last layer of the pretrained neural network (see Fig. 3). The final stage of the vector extraction process from the ResNet-50 network was the average pooling. To obtain information on whether the recorded image is an anomaly, the vector is passed to the AD block (see Fig. 1).

**Fig. 5:**
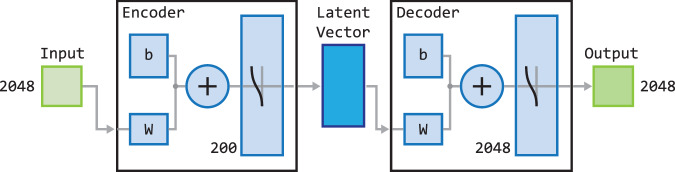
Diagram of the Autoencoder used in the anomaly detection method.

**Fig. 6:**
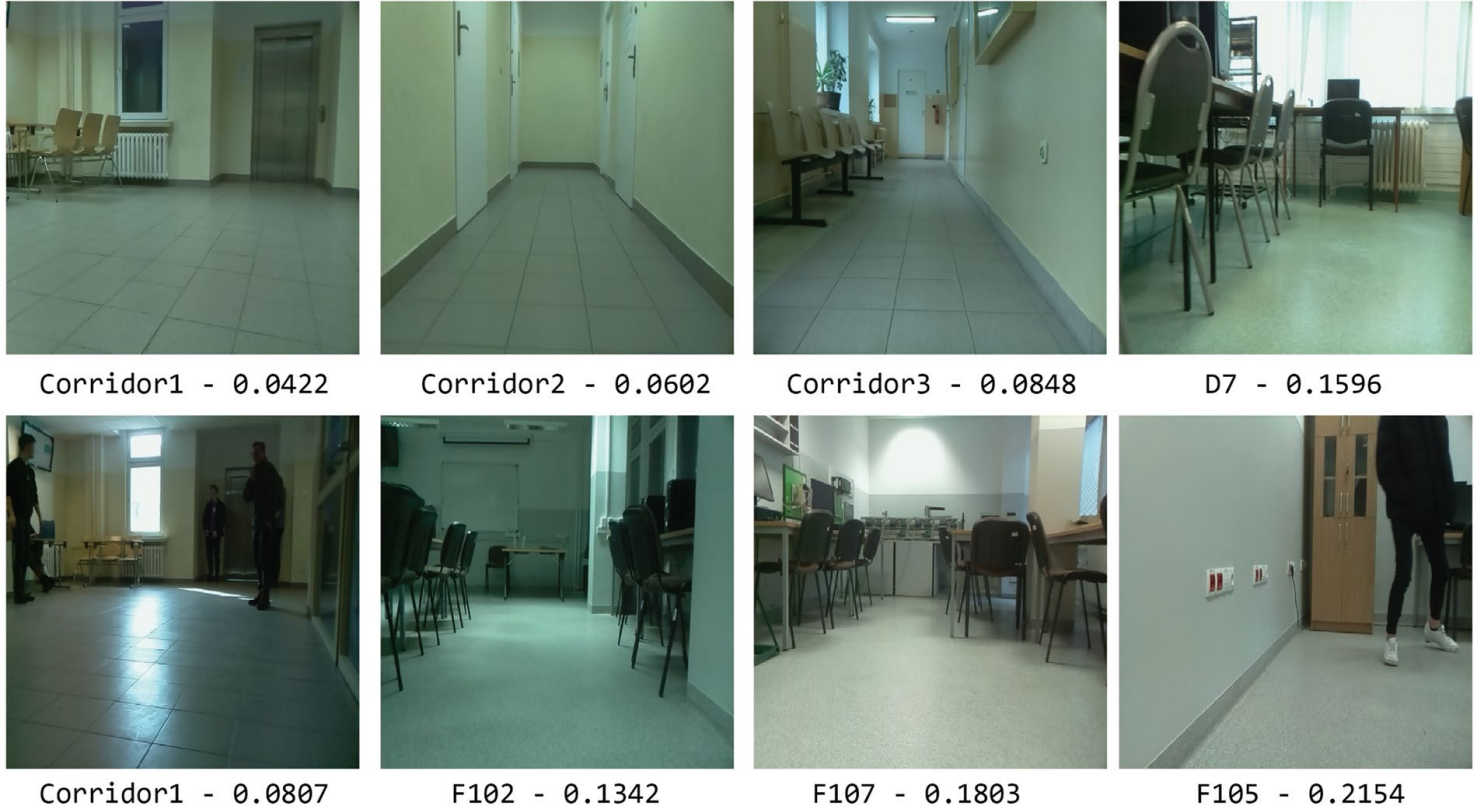
Example cosine distance values for Anomaly detection based on Autoencoder with known Corridor1 and other unknown rooms.

Figure 5 shows a detailed diagram of the Autoencoder. Each of the Autoencoders implemented for the experiments was based on the same training parameters. The sigmoid activation function was used in the output layer of the Autoencoder for training. An Autoencoder consisting of a single hidden layer with 200 neurons was trained in 1000 epochs. When the AE implements this block, the input vector is processed by the Encoder, resulting in a hidden representation (Latent Vector). This hidden representation is then utilized by the Decoder. Finally, the difference between the original feature vector (${\bf{x}}$) and the reconstructed vector (${\bf{x}}{\rm{{\prime} }}$) is treated as the result of the trained AE and cosine distance (1). Figure 6 shows the sample results for the feature reconstruction-based method. The results are based solely on the data only for room “Corridor1”. The remaining rooms are anomalies in terms of the trained Autoencoder. The values below the images in the figure indicate the distance between the original feature vector and the reconstructed one. The value is compared to a global threshold that determines whether the analyzed data is an anomaly. The image shows that the result is influenced by the different appearance of the image, i.e. lighting conditions, arrangement of objects, and the pose of the camera. The lowest result was achieved on an image of a known room with favorable conditions. The highest was given another room with human activity. The figure also shows that changing conditions in the scene can cause the method to consider the room as unknown. The problem is that the emerging anomalies significantly affect the operation of the method. The purpose of testing the method based on the description of the entire image is to test whether it will work well in the case of local disturbances. The examples given at the beginning present room recognition results influenced by the occurring anomalies. The topic will be discussed in more detail in next sections.

## Data Records

**Fig. 7:**
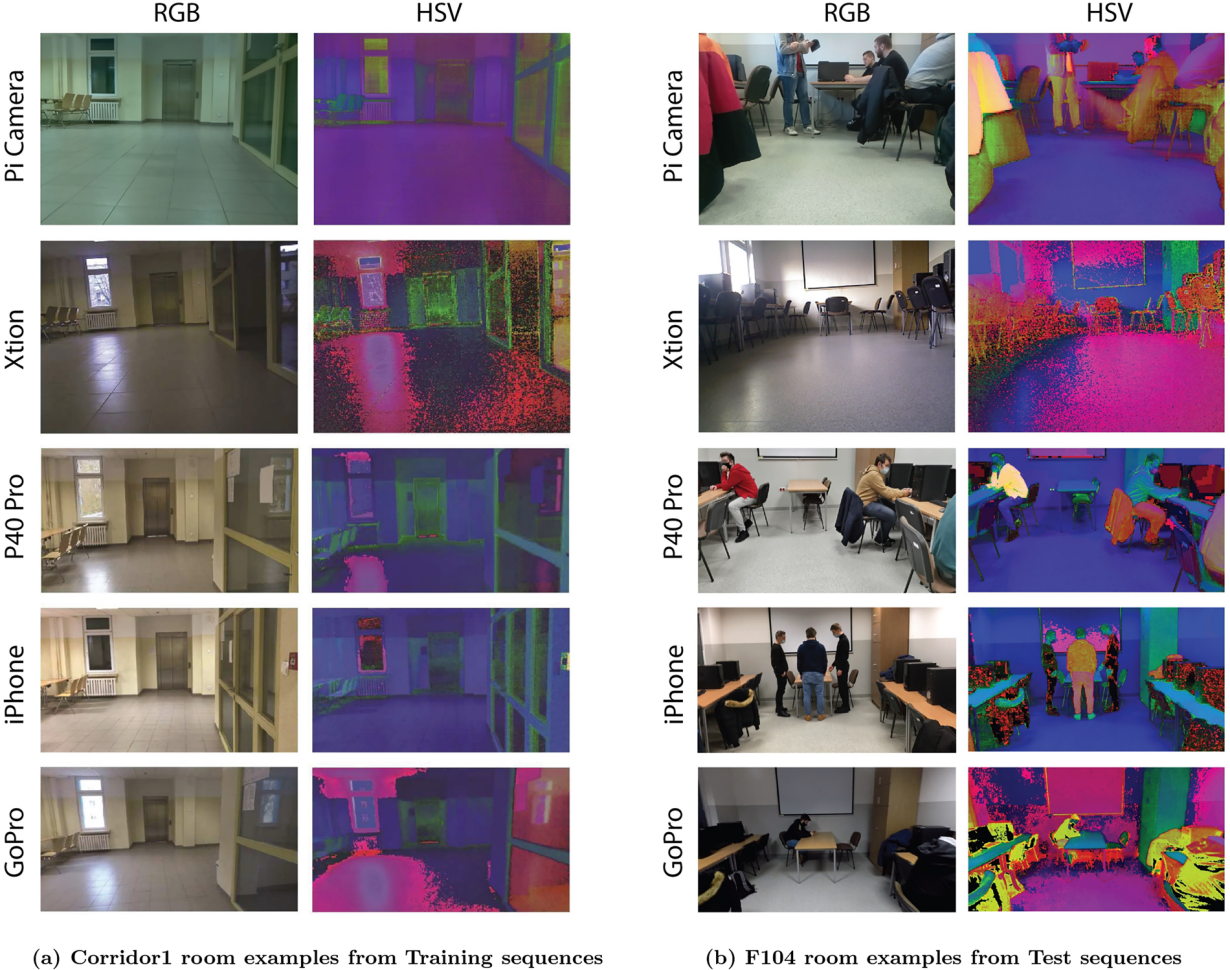
Example visualization of the dataset images.

The proposed dataset has been made publicly available under the CC BY 4.0 license and can be downloaded from the ZENODO repository^90^ The data are divided into five sets (containing data for different cameras), which have further subsets. Each of the subsets: Training, Test 1, Test 2, and Test 3 consists of nine image sequences. Detailed information on the data collection is presented in Table 2. A total of 89,550 three-channel RGB color images in PNG format are organized into 20 zip folders with a whole size of 34.3 GB. Each image in the sequence has a label that represents a room. The number of images for each subset differs due to the split into training and testing data. The difference also results from different methods of recording the image sequences. In order to have balanced data in the subsets, each room in the sequence has the same number of images. Different environmental changes were introduced in each subset. The data from Test 1 are closest to those from the training set. The differences between the sequences are mainly due to changes in the route, robot, and recording equipment. The rooms are well lighted, but not overexposed. The sequences from Test 3 present changed conditions, such as a different time of day, a changed lighting system, and intensive layout changes. The key change is the different paths of the human and the robot. This means a different perspective from previously recorded scenes. The Test 2 sequences pose the most difficult challenge because they contain various recorded activities performed by people moving around rooms. People can occlude important parts of the scene and pass in front of the camera. The images were anonymized by manually blurring the faces of observed people. An example of photos taken for the collection is shown in Fig. 7. The examples shown are represented in the RGB and HSV color spaces. This allowed us to notice differences between the proposed sets at the pixel level. Each camera had different properties (e.g. resolution, field of view, sensor type) and their settings were not interfered with to highlight the different recording devices. The dataset consists of independent image sequences recorded during separate camera passes, with each pass captured by only one camera. As a result, there was no necessity for synchronization between different cameras within the entire dataset. The data collection process involved two separate systems. The first system was a mobile platform based on a Raspberry Pi v2 equipped with a PiCamera OV5647, running Python version 2.7. The Raspberry Pi v2 featured a 900 MHz ARM Cortex-A7 CPU (quad-core) and 1GB RAM, operating on Raspbian OS. The Raspberry Pi was connected to a laptop enclosed within the robot, serving as the data collection unit. The second system utilized a NAO Robot V4 manufactured by Aldebaran Robotics with OpenNAO software, which worked in collaboration with the Xtion camera. This robot was powered by an Intel Atom Z530 @ 1.6 GHz processor, 1 GB RAM, and 8 GB Flash memory. Data acquisition on this platform was conducted using Python 2.7 for compatibility with the Nao robot’s software environment. Additionally, for data registration with the iPhone, P40 Pro, and GoPro, default recording settings were applied according to the specifications provided in Table 2.

**Table 2:** Characteristics of image and number for individual sets, subsets, and sequences.

Subset	Mounted	Total images (Images per place)				Image properties		
		Training	Test 1	Test 2	Test 3	Width (px)	Height (px)	Aspect ratio
Pi Camera	Robot	7,200 (800)	5,400 (600)	5,400 (600)	5,400 (600)	640	480	1.3
Xtion	Robot	7,200 (800)	1,800 (200)	1,800 (200)	1,800 (200)	640	480	1.3
GoPro	Hand	5,400 (600)	4,500 (500)	4,500 (500)	4,500 (500)	960	540	1.7
iPhone	Hand	5,400 (600)	4,500 (500)	4,500 (500)	4,500 (500)	960	540	1.7
P40Pro	Hand	5,400 (600)	4,050 (450)	3,150 (350)	3,150 (350)	640	360	1.7

**Fig. 8:**
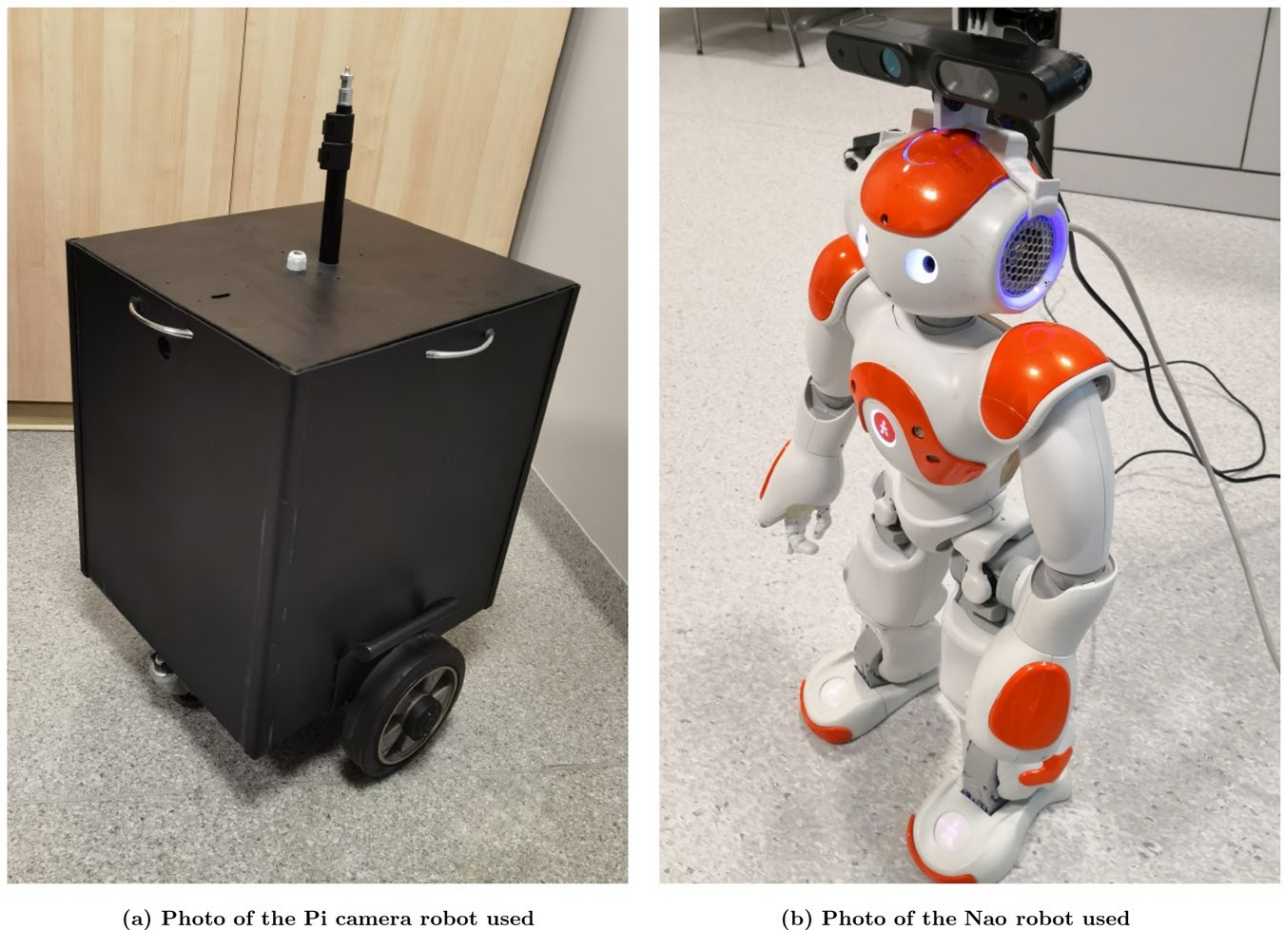
Images depicting the mobile robots used.

Part of the prepared data were used in previous research on the multi-domain classification problem^71^. This data has been updated with a new set recorded using a wheeled robot. As a result, a comprehensive and extended dataset was developed, which constitutes the basis for further research. It was important to adapt the set to problems such as visual location recognition or anomaly detection. The proposed dataset is divided into training and testing data based on independent recordings in rooms. This is consistent with a real scenario in which the camera registers rooms with changes in the environment that occur naturally (e.g. changes in lighting, change in the position of equipment, presence of people). The images of the three sets were collected using a hand-made recording. The devices used included the GoPro Hero 5 Black, iPhone 6s, and HUAWEI P40 Pro. Two types of robots (wheeled and humanoid) were used for the remaining sets. A custom wheeled robot (Fig. 8) had a Pi camera mounted on the front. The camera placed behind the robot’s cover was approximately 60 cm above the ground. The robot is designed to travel indoors and moves at a maximum speed of 1 m/sec. The second robot used is a Nao humanoid robot and had an ASUS Xtion PRO LIVE camera mounted on its head^62^, see Figure 8. When registering the dataset, the focus was on the data that represented real scenes as closely as possible. The rooms were used normally and were not adapted in any way for experiments. Additionally, the registration was done in on various conditions (e.g. illumination) that may occur in selected rooms. Examples include changes in lighting, different outdoor conditions, and the movement and activity of people. The shared resources are not limited to one device and recording method. Five cameras with different parameters and two robots of different types were used. Data collected during human and robot movement were combined in one dataset. The goal was to collect more diverse data from the same environment. This is especially important when implementing swarms of robots that may differ in camera location and movement.

Main features of the developed benchmark dataset:

- nine indoor places, each represented by 9,950 color images (total 89,550 images);
- data collected by robots and manually by humans;
- various types of mobile robots (humanoid, wheeled);
- five different cameras with different lenses and parameters;
- registration of rooms with varied conditions and time intervals;
- sequences with the dynamic activity of anonymous people on stage;
- the high complexity of the collection with different conditions, ways of recording and moving the camera.

## Technical Validation

**Fig. 9:**
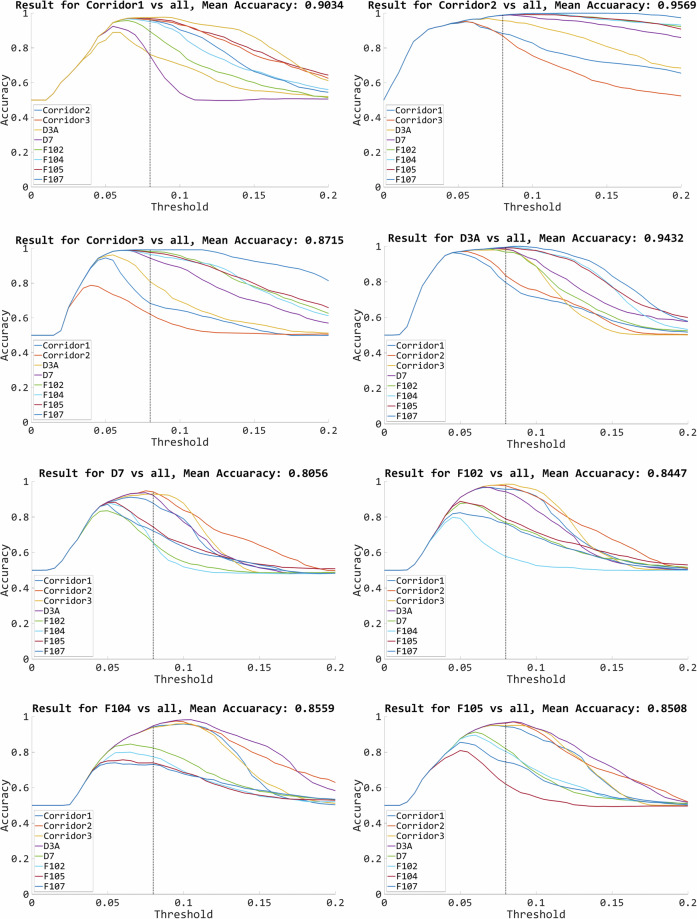
Anomaly detection accuracy diagrams for different rooms and variable thresholds. Autoencoder, Pi Camera (Test 1).

The main result of the experiments was the recognition of the room. A one-vs-one approach was performed in which a new test subset was created from the selected test sequences. The new test subset consists of data from two places (known room and unknown room). This allowed for balancing the data, so the smaller number of outlier samples did not influence the final result. In a real environment, this is a common situation where outlier samples are rare and data mainly contain normal samples. This limits the possibility of reliably checking the room recognition methods. To train the evaluated methods, data from the training subset were used. A total of 225 tests were performed, which led to the final result. In the experiments, the methods discussed in the section “Methods” were evaluated. For the purposes of the study, the thresholds for each detection method were selected and used in all tests. The global threshold values for IF, OCSVM, and AE methods are 0.45, -0.2, and 0.08, respectively. These values were determined experimentally based on the analysis of the results of test sequences of a subset of the Pi Camera. Examples of the analyzed charts are shown in Fig. 9. In each of the remaining tests, depending on the method utilized, an identical global threshold was used. The following sections provide tables with the experimental results obtained in the evaluations discussed above. There is a division depending on the use of a single image and a sequence. The final section also shows the aggregated results for all tests. This allowed us to determine the average precision of the tested methods.

### Single-image experiments

Tables 3, 4, and 5 contain example results of anomaly detection. The first column contains the name of the room used in training the anomaly detection method. In all experiments, only images from the training sequence were used in the training of the model. This means that the training and testing data are separated by the structure of the proposed sets. The first row of the tables shows rooms in the test sets that are anomalies. The single result is for test images of a known room (first column) and an unknown room (first row). This approach allowed us to check the characteristics of the data for the recorded rooms. The result shows which subsets are demanding and which rooms are visually closer. Table 3 shows the Isolation Forest accuracy for the Pi Camera Test 1 set. The lowest anomaly detection result was obtained with this method for pair F104 and F107 (0.5425). The arithmetic mean is 0.7520 and the standard deviation (std. dev.) is 0.1004. Tables 4 and 5 show results for OCSVM (mean – 0.7737, std. dev. – 0.1545) and AE (mean – 0.8744, std. dev. – 0.1204).

**Table 3:** Anomaly detection accuracy achieved by Isolation Forest, Pi Camera (Test 1).

	Corridor1	Corridor2	Corridor3	D3	D7	F102	F104	F105	F107
**Corridor1**	—	0.7517	0.7183	0.7683	0.7108	0.8842	0.8242	0.7800	0.7467
**Corridor2**	0.7483	—	0.7125	0.8725	0.7683	0.8842	0.8217	0.8092	0.7825
**Corridor3**	0.7883	0.7525	—	0.9433	0.8342	0.9233	0.8175	0.8408	0.8217
**D3**	0.7292	0.7525	0.7175	—	0.6292	0.7100	0.7983	0.6892	0.6733
**D7**	0.8033	0.7525	0.7875	0.9208	—	0.6725	0.7217	0.6250	0.6358
**F102**	0.8283	0.7525	0.7875	0.9158	0.6192	—	0.6833	0.6567	0.5808
**F104**	0.8158	0.7525	0.7817	0.9483	0.5825	0.5867	—	0.5758	0.5425
**F105**	0.8292	0.7525	0.7842	0.9458	0.5800	0.6825	0.6233	—	0.5992
**F107**	0.8333	0.7525	0.7892	0.9567	0.6658	0.6967	0.6575	0.6600	—

**Table 4:** Anomaly detection accuracy achieved by One-Class Support Vector Machine, Pi Camera (Test 1).

	Corridor1	Corridor2	Corridor3	D3	D7	F102	F104	F105	F107
**Corridor1**	—	0.7600	0.6783	0.7183	0.7942	0.9542	0.9883	0.6692	0.8458
**Corridor2**	0.7075	—	0.6717	0.7617	0.9050	0.9775	0.9842	0.8475	0.9483
**Corridor3**	0.4925	0.5750	—	0.8108	0.7400	0.9558	0.9725	0.6117	0.7608
**D3**	0.7508	0.9125	0.7875	—	0.7975	0.8708	0.9817	0.7150	0.8175
**D7**	0.7875	0.9517	0.9217	0.7917	—	0.6450	0.7433	0.5333	0.6208
**F102**	0.9133	0.9600	0.9750	0.8067	0.5883	—	0.7483	0.5542	0.5767
**F104**	0.8067	0.9575	0.9217	0.8517	0.5000	0.5083	—	0.4967	0.4892
**F105**	0.8633	0.9467	0.9350	0.9208	0.6117	0.6908	0.6733	—	0.6058
**F107**	0.9025	0.9600	0.9783	0.8867	0.5492	0.6075	0.6275	0.5317	—

**Table 5:** Anomaly detection accuracy achieved by Autoencoder, Pi Camera (Test 1).

	Corridor1	Corridor2	Corridor3	D3	D7	F102	F104	F105	F107
**Corridor1**	—	0.7625	0.7550	0.8942	0.9508	0.9683	0.9608	0.9617	0.9742
**Corridor2**	0.8833	—	0.8700	0.9575	0.9875	0.9892	0.9892	0.9892	0.9892
**Corridor3**	0.6833	0.6217	—	0.8083	0.9433	0.9817	0.9650	0.9767	0.9917
**D3**	0.7958	0.8350	0.9817	—	0.9850	0.9675	0.9942	0.9917	0.9950
**D7**	0.8775	0.9417	0.9292	0.9225	—	0.6550	0.6533	0.7442	0.7217
**F102**	0.9558	0.9758	0.9850	0.9425	0.7683	—	0.5775	0.7892	0.7633
**F104**	0.9425	0.9483	0.9400	0.9483	0.8233	0.7733	—	0.7400	0.7317
**F105**	0.9433	0.9633	0.9475	0.9658	0.8192	0.8033	0.6200	—	0.7442
**F107**	0.9683	0.9675	0.9692	0.9667	0.8183	0.6667	0.5767	0.7683	—

The method established global thresholds for individual methods. This was a condition for the final decision on the recognition of data as anomalies. Figure 9 shows the sample results for various threshold values for the Test 1 sequence from the Pi Camera collection. The diagrams compare knowledge about one room with the rest, being treated as anomalies. Based on the graphs presented, it can be concluded that for some rooms, the lower results are due to the adopted way of selecting the optimal threshold. The threshold for each method was selected on the basis of the training data, but it does not have to be the optimal value for all data. The diagrams also show that some rooms in the collection are visually different from others. This means that it is easier to get a higher score regardless of the threshold. In these cases, when the threshold changes, the accuracy line is parallel to the abscissa. The presence of a hill means that the selection of the threshold has a greater impact on the final result. Analyses based on knowledge about the threshold and the result also show that for some places the image description from the network and the method do not provide sufficient recognition accuracies. The similarities between rooms may be too great and the anomaly detection mechanism should be more sensitive to details in the image.

### Multi-image experiments

The second experiment consists of using image sequences to detect an unknown room. According to the order in which the images were taken, the result was obtained for different subsequence sizes, i.e. images in a sequence. The method of aggregation of the results was the median of the results according to the scheme presented in Fig. 2. Tabels 5, 6, 7, 8, and 9 present the results for changing the size of the used subsequence: 1, 5, 10, 25, all images from the room (all). The results are based on an approach in which a robot that moves around rooms can make decisions based on previous recorded images. This allows to improve the recognition accuracy in some ambiguous situations. Table 9 shows results in which the decision about the anomaly is made based on all images from the room. Increasing the number of images generally improves the final result. However, in some cases, the results deteriorate (e.g. D7 vs. F102).

**Table 6:** Anomaly detection accuracy for Autoencoder, Pi Camera (Test 1) with five images in subsequence.

	Corridor1	Corridor2	Corridor3	D3	D7	F102	F104	F105	F107
**Corridor1**	—	0.7583	0.7750	0.9042	0.9625	0.9792	0.9667	0.9708	0.9792
**Corridor2**	0.8833	—	0.8708	0.9542	0.9875	0.9875	0.9875	0.9875	0.9875
**Corridor3**	0.6792	0.6208	—	0.8083	0.9458	0.9833	0.9667	0.9792	0.9917
**D3**	0.8000	0.8375	0.9833	—	0.9917	0.9750	0.9958	0.9917	0.9958
**D7**	0.8917	0.9458	0.9292	0.9333	—	0.6542	0.6333	0.7500	0.7083
**F102**	0.9667	0.9875	0.9917	0.9500	0.7583	—	0.5875	0.8042	0.7667
**F104**	0.9458	0.9500	0.9417	0.9500	0.8292	0.7708	—	0.7458	0.7333
**F105**	0.9542	0.9708	0.9500	0.9708	0.8333	0.8083	0.6125	—	0.7458
**F107**	0.9750	0.9750	0.9750	0.9750	0.8250	0.6667	0.5750	0.7667	—

**Table 7:** Anomaly detection accuracy for Autoencoder, Pi Camera (Test 1) using 10 images in subsequence.

	Corridor1	Corridor2	Corridor3	D3	D7	F102	F104	F105	F107
**Corridor1**	—	0.7583	0.7750	0.9083	0.9667	0.9750	0.9667	0.9667	0.9750
**Corridor2**	0.9000	—	0.8750	0.9750	1.0000	1.0000	1.0000	1.0000	1.0000
**Corridor3**	0.6833	0.6250	—	0.8083	0.9667	0.9833	0.9750	0.9833	0.9917
**D3**	0.8083	0.8583	0.9833	—	1.0000	0.9667	1.0000	1.0000	1.0000
**D7**	0.8667	0.9500	0.9250	0.9417	—	0.6417	0.6417	0.7250	0.7000
**F102**	0.9667	0.9917	0.9917	0.9417	0.7667	—	0.6000	0.7917	0.7583
**F104**	0.9583	0.9583	0.9500	0.9583	0.8333	0.7833	—	0.7250	0.7500
**F105**	0.9583	0.9750	0.9667	0.9750	0.8333	0.8167	0.6167	—	0.7583
**F107**	0.9667	0.9667	0.9667	0.9667	0.8167	0.6417	0.5667	0.7833	—

**Table 8:** Anomaly detection accuracy for Autoencoder, Pi Camera (Test 1) using 25 images in subsequence.

	Corridor1	Corridor2	Corridor3	D3	D7	F102	F104	F105	F107
**Corridor1**	—	0.7500	0.7708	0.9375	0.9792	0.9792	0.9792	0.9792	0.9792
**Corridor2**	0.8958	—	0.8958	0.9792	1.0000	1.0000	1.0000	1.0000	1.0000
**Corridor3**	0.7083	0.6250	—	0.8125	0.9792	1.0000	1.0000	1.0000	1.0000
**D3**	0.7917	0.8542	0.9792	—	1.0000	0.9792	1.0000	1.0000	1.0000
**D7**	0.8750	0.9583	0.9375	0.9583	—	0.6458	0.6458	0.7708	0.7083
**F102**	0.9792	1.0000	1.0000	0.9583	0.7917	—	0.5833	0.8333	0.7708
**F104**	0.9583	0.9583	0.9583	0.9583	0.8333	0.7917	—	0.7500	0.7292
**F105**	0.9583	0.9792	0.9583	0.9792	0.8542	0.8542	0.6042	—	0.7708
**F107**	0.9792	0.9792	0.9792	0.9792	0.8333	0.6875	0.6042	0.7917	—

**Table 9:** Anomaly detection accuracy for Autoencoder, Pi Camera (Test 1) using all images.

	Corridor1	Corridor2	Corridor3	D3	D7	F102	F104	F105	F107
**Corridor1**	—	1.0000	1.0000	1.0000	1.0000	1.0000	1.0000	1.0000	1.0000
**Corridor2**	1.0000	—	1.0000	1.0000	1.0000	1.0000	1.0000	1.0000	1.0000
**Corridor3**	0.5000	0.5000	—	1.0000	1.0000	1.0000	1.0000	1.0000	1.0000
**D3**	1.0000	1.0000	1.0000	—	1.0000	1.0000	1.0000	1.0000	1.0000
**D7**	1.0000	1.0000	1.0000	1.0000	—	0.5000	0.5000	1.0000	1.0000
**F102**	1.0000	1.0000	1.0000	1.0000	1.0000	—	0.5000	1.0000	1.0000
**F104**	1.0000	1.0000	1.0000	1.0000	1.0000	1.0000	—	1.0000	1.0000
**F105**	1.0000	1.0000	1.0000	1.0000	1.0000	1.0000	0.5000	—	1.0000
**F107**	1.0000	1.0000	1.0000	1.0000	1.0000	0.5000	0.5000	1.0000	—

### Aggregated result

Results for the single and multi-image approaches were aggregated into a single output table. Table 10 shows the mean accuracy for the Isolation Forest, One-Class Support Vector Machine, and Autoencoder methods. The best results in the table are shown in bold. The division of the table takes into account results on five subsets: Pi Camera, Xtion, GoPro, iPhone, and P40 Pro. Five image sequence sizes were considered: 1, 5, 10, 25, and all. The size of the “all” variant varies depending on the size of the test sequence. This table shows the characteristics of the methods and the effect of changing the number of images in the test based on mean and standard deviation. The last column shows the average results of all experiments for individual subsets. Based on this parameter, conclusions can be drawn about how difficult a given test subset is. The highest accuracy was achieved in the Test 1 sequences, which had a limited amount of noise. The recorded images were similar to those during training and therefore had a lower difficulty level than other test subsets. The average accuracy is lower in the Test 3 sequences, where changes have been made to the lighting and environment. The smallest recognition accuracies were obtained for Test 2. This is due to the specificity of the data and the occurrence of large changes in the images, e.g. human activity. Based on the arithmetic mean, the presence of people in the scene causes a significant deterioration of the performance of the tested methods. In the context of using image sequences, depending on the variant, it is possible to worsen or improve the individual test result. However, in terms of average, an improvement can be observed. The conclusion is that in this case, it is worth relying on a subsequence of images from the analyzed rooms. As the number of images used increases, the recognition accuracy also increases. The results also show that with the selected thresholds, the final highest accuracy was achieved with the Autoencoder-based method.

**Table 10:** Average accuracy of the methods for the dataset.

Subsequence size		1			5			10			25			all			Mean
Training	Test	IF	OCSVM	AE	IF	OCSVM	AE	IF	OCSVM	AE	IF	OCSVM	AE	IF	OCSVM	AE	
Pi Camera	Test 1	0.7520 ± 0.100	0.7737 ± 0.154	**0.8744** ± **0.120**	0.7568 ± 0.104	0.7748 ± 0.158	**0.8777** ± **0.122**	0.7634 ± 0.105	0.7774 ± 0.159	**0.8801** ± **0.125**	0.7688 ± 0.104	0.7841 ± 0.167	**0.8889** ± **0.123**	0.9028 ± 0.198	0.8125 ± 0.242	**0.9444** ± **0.157**	0.8221
	Test 2	0.6005 ± 0.081	0.7076 ± 0.106	**0.7090** ± **0.086**	0.5992 ± 0.081	**0.7158** ± **0.108**	0.7116 ± 0.085	0.6008 ± 0.084	**0.7133** ± **0.114**	0.7061 ± 0.092	0.6100 ± 0.098	**0.7260** ± **0.121**	0.7205 ± 0.111	0.6111 ± 0.208	**0.7986** ± **0.247**	0.7222 ± 0.248	0.6835
	Test 3	0.7183 ± 0.123	0.7613 ± 0.151	**0.8447** ± **0.106**	0.7275 ± 0.128	0.7665 ± 0.151	**0.8476** ± **0.105**	0.7308 ± 0.126	0.7669 ± 0.156	**0.8542** ± **0.106**	0.7459 ± 0.133	0.7775 ± 0.162	**0.8634** ± **0.107**	0.8264 ± 0.238	0.8194 ± 0.240	**0.9514** ± **0.148**	0.8001
Xtion	Test 1	0.7181 ± 0.129	0.7398 ± 0.151	**0.8632** ± **0.129**	0.6970 ± 0.151	**0.8727** ± **0.161**	**0.8727** ± **0.134**	0.7399 ± 0.152	0.7469 ± 0.168	**0.8861** ± **0.136**	0.7656 ± 0.175	0.7604 ± 0.183	**0.9022** ± **0.139**	0.7986 ± 0.245	0.7778 ± 0.248	**0.9028** ± **0.198**	0.7945
	Test 2	0.5790 ± 0.086	0.6699 ± 0.108	**0.6815** ± **0.089**	0.5802 ± 0.098	0.6859 ± 0.116	**0.6925** ± **0.092**	0.5858 ± 0.101	**0.7073** ± **0.124**	0.6969 ± 0.103	0.5929 ± 0.119	**0.7179** ± **0.149**	0.7144 ± 0.130	0.6319 ± 0.250	**0.7222** ± **0.248**	**0.7222** ± **0.248**	0.6654
	Test 3	0.6981 ± 0.135	0.7361 ± 0.170	**0.7735** ± **0.157**	0.7161 ± 0.152	0.7468 ± 0.176	**0.7794** ± **0.163**	0.7441 ± 0.152	0.7608 ± 0.186	**0.7806** ± **0.175**	0.7465 ± 0.183	0.7674 ± 0.190	**0.8038** ± **0.160**	**0.8333** ± **0.236**	0.8056 ± 0.244	0.7917 ± 0.298	0.7656
GoPro	Test 1	0.7885 ± 0.104	0.8851 ± 0.092	**0.9192** ± **0.068**	0.8052 ± 0.109	0.8867 ± 0.092	**0.9240** ± **0.065**	0.8151 ± 0.110	0.8931 ± 0.091	**0.9346** ± **0.059**	0.8455 ± 0.112	0.9108 ± 0.084	**0.9372** ± **0.066**	0.9583 ± 0.138	0.9722 ± 0.115	**0.9931** ± **0.059**	0.8979
	Test 2	0.6097 ± 0.055	**0.7110** ± **0.084**	0.6838 ± 0.083	0.6091 ± 0.054	**0.7141** ± **0.080**	0.6856 ± 0.084	0.6113 ± 0.059	**0.7193** ± **0.094**	0.6851 ± 0.086	0.6146 ± 0.061	**0.7194** ± **0.093**	0.6760 ± 0.090	0.5556 ± 0.157	**0.7778** ± **0.248**	0.6667 ± 0.236	0.6693
	Test 3	0.7693 ± 0.106	0.8126 ± 0.159	**0.8189** ± **0.185**	0.7791 ± 0.112	0.8196 ± 0.161	**0.8248** ± **0.189**	0.7849 ± 0.117	0.8242 ± 0.168	**0.8257** ± **0.190**	0.8323 ± 0.118	**0.8427** ± **0.170**	0.8309 ± 0.194	**0.9236** ± **0.180**	0.8681 ± 0.236	0.8819 ± 0.212	0.8292
iPhone	Test 1	0.7234 ± 0.112	0.8025 ± 0.133	**0.8588** ± **0.064**	0.7380 ± 0.120	0.8099 ± 0.137	**0.8697** ± **0.062**	0.7522 ± 0.129	0.8131 ± 0.144	**0.8715** ± **0.061**	0.7660 ± 0.120	0.8174 ± 0.152	**0.8979** ± **0.065**	0.8681 ± 0.220	0.8542 ± 0.227	**1.0000** ± **0.000**	0.8295
	Test 2	0.6185 ± 0.084	**0.7248** ± **0.108**	0.6702 ± 0.095	0.6219 ± 0.089	**0.7309** ± **0.111**	0.6713 ± 0.093	0.6194 ± 0.093	**0.7301** ± **0.114**	0.6722 ± 0.098	0.6358 ± 0.081	**0.7354** ± **0.123**	0.6639 ± 0.085	0.6528 ± 0.245	**0.7778** ± **0.248**	0.5556 ± 0.157	0.6720
	Test 3	0.7294 ± 0.122	0.7930 ± 0.156	**0.8009** ± **0.177**	0.7457 ± 0.130	0.7984 ± 0.162	**0.8074** ± **0.182**	0.7583 ± 0.142	0.8085 ± 0.160	**0.8129** ± **0.185**	0.7663 ± 0.145	0.8215 ± 0.172	**0.8299** ± **0.193**	0.8750 ± 0.217	0.8750 ± 0.217	**0.8889** ± **0.208**	0.8074
P40Pro	Test 1	0.7807 ± 0.135	0.7935 ± 0.151	**0.9000** ± **0.092**	0.8000 ± 0.138	0.8015 ± 0.160	**0.9026** ± **0.093**	0.8184 ± 0.141	0.8015 ± 0.165	**0.9147** ± **0.088**	0.8461 ± 0.145	0.8198 ± 0.183	**0.9275** ± **0.094**	0.9236 ± 0.180	0.8333 ± 0.236	**0.9861** ± **0.082**	0.8566
	Test 2	0.5860 ± 0.081	**0.6781** ± **0.115**	0.6272 ± 0.066	0.5842 ± 0.084	**0.6828** ± **0.126**	0.6243 ± 0.067	0.5948 ± 0.086	**0.6813** ± **0.130**	0.6242 ± 0.069	0.6066 ± 0.089	**0.6954** ± **0.150**	0.6270 ± 0.080	0.6042 ± 0.203	**0.7292** ± **0.249**	0.5000 ± 0.000	0.6297
	Test 3	0.7609 ± 0.108	0.7760 ± 0.152	**0.8807** ± **0.078**	0.7754 ± 0.116	0.7826 ± 0.158	**0.8900** ± **0.082**	0.7982 ± 0.117	0.7968 ± 0.166	**0.9042** ± **0.076**	0.8219 ± 0.137	0.7976 ± 0.175	**0.9082** ± **0.085**	0.9167 ± 0.186	0.8264 ± 0.238	**1.0000** ± **0.000**	0.8424
	Mean	0.7038	0.7639	**0.8018**	0.7111	0.7788	**0.8063**	0.7237	0.7738	**0.8109**	0.7409	0.7840	**0.8198**	0.8036	0.8234	**0.8418**	
	Std. dev.	0.0659	0.0495	0.0876	0.0716	0.0547	0.0899	0.0758	0.0509	0.0941	0.0824	0.0530	0.0984	0.1284	0.0542	0.1571	

## Usage Notes

The MDDRobots dataset is a collection of image sequences captured between 2019 and 2024. The data package is available for use under the CC BY 4.0 license and provided by the ZENODO repository^90^ The dataset is a set of sequences of RGB images taken by moving cameras, including those mounted on robots. These images depict the interior of a building located on the university campus of Rzeszow University of Technology. Various cameras were used to capture the images recorded the surroundings from various angles and under different environmental conditions. The dataset has been carefully prepared and organized to facilitate research on VPR and anomaly detection. The number of images in the dataset has been determined to ensure a balanced distribution across different sets. This dataset poses challenges for a variety of research topics. It contains real-world situations that may be problematic for using robots with onboard cameras. The presented data can be a basis for many works for better understanding VPR and anomaly detection in multi-domain environments. It fills gaps that occur in more sterile and laboratory-prepared collections that lack a multi-domain representation of places. An important aspect of the dataset are recordings containing people. In these cases, face anonymization was performed by manually blurring the faces.

## Data Availability

An example of the code handling the dataset is publicly available on GitHub https://github.com/WozniakP/MDDRobots. To implement methods and conduct experiments, the MATLAB (version 2021a) environment was used. The shared repository contains information about the requirements and libraries needed to run a demo for anomaly detection. The links to the dataset provided in the article contain the trained neural models from which the results were obtained.

## References

[1] Savkin, A. V. & Wang, C. Seeking a path through the crowd: Robot navigation in unknown dynamic environments with moving obstacles based on an integrated environment representation. *Robotics and Autonomous Systems* **62**, 1568–1580, 10.1016/j.robot.2014.05.006 (2014).

[2] Tsai, G. & Kuipers, B. Dynamic visual understanding of the local environment for an indoor navigating robot. In *IEEE/RSJ Int. Conf. on Intelligent Robots and Systems*, 4695–4701, 10.1109/IROS.2012.6385735 (2012).

[3] Alqobali, R., Alnasser, R., Rashidi, A., Alshmrani, M. & Alhmiedat, T. A Real-Time Semantic Map Production System for Indoor Robot Navigation. *Sensors* **24**, 10.3390/s24206691 (2024). 10.3390/s24206691 PMC11511299 39460171

[4] Sahdev, R., Chen, B. X. & Tsotsos, J. K. Indoor Localization in Dynamic Human Environments Using Visual Odometry and Global Pose Refinement. In *15th Conf. on Computer and Robot Vision (CRV)*, 360–367, 10.1109/CRV.2018.00057 (2018).

[5] Luo, J., Pronobis, A., Caputo, B. & Jensfelt, P. Incremental learning for place recognition in dynamic environments. In *IEEE/RSJ Int. Conf. on Intelligent Robots and Systems*, 721–728, 10.1109/IROS.2007.4398986 (2007).

[6] Peng, G. *et al*. TransLoc4D: Transformer-based 4D Radar Place Recognition. In *Proc. of the IEEE/CVF Conf. on Computer Vision and Pattern Recognition (CVPR)*, 17595–17605, 10.1109/CVPR52733.2024.01666 (2024).

[7] Meng, C. *et al*. mmPlace: Robust Place Recognition With Intermediate Frequency Signal of Low-Cost Single-Chip Millimeter Wave Radar. *IEEE Robotics and Automation Letters* **9**, 4878–4885, 10.1109/LRA.2024.3377562 (2024).

[8] Kim, G., Park, Y. S., Cho, Y., Jeong, J. & Kim, A. MulRan: Multimodal Range Dataset for Urban Place Recognition. In *IEEE Int. Conf. on Robotics and Automation (ICRA)*, 6246–6253, 10.1109/ICRA40945.2020.9197298 (2020).

[9] Lowry, S. *et al*. Visual Place Recognition: A Survey. *IEEE Trans. on Robotics* **32**, 1–19, 10.1109/TRO.2015.2496823 (2015).

[10] Schubert, S., Neubert, P., Garg, S., Milford, M. & Fischer, T. Visual Place Recognition: A Tutorial. *IEEE Robotics & Automation Magazine* 2–16, 10.1109/MRA.2023.3310859 (2023).

[11] Kannan, S. S. & Min, B.-C. PlaceFormer: Transformer-Based Visual Place Recognition Using Multi-Scale Patch Selection and Fusion. *IEEE Robotics and Automation Letters* **9**, 6552–6559, 10.1109/LRA.2024.3408075 (2024).

[12] Cui, L. & Ma, C. SOF-SLAM: A Semantic Visual SLAM for Dynamic Environments. *IEEE Access* **7**, 166528–166539, 10.1109/ACCESS.2019.2952161 (2019).

[13] Tsintotas, K. A., Bampis, L. & Gasteratos, A. The revisiting problem in simultaneous localization and mapping: A survey on visual loop closure detection. *IEEE Trans. on Intelligent Transportation Systems* **23**, 19929–19953, 10.1109/TITS.2022.3175656 (2022).

[14] Tee, M. K. T., Lau, B. & Siswoyo Jo, H. An Improved Indoor Robot Human-Following Navigation Model Using Depth Camera, Active IR Marker and Proximity Sensors Fusion. *Robotics* **7**, 10.3390/robotics7010004 (2018).

[15] Ji, T., Sivakumar, A. N., Chowdhary, G. & Driggs-Campbell, K. Proactive Anomaly Detection for Robot Navigation With Multi-Sensor Fusion. *IEEE Robotics and Automation Letters* **7**, 4975–4982, 10.1109/LRA.2022.3153989 (2022).

[16] Yuan, W., Li, Z. & Su, C.-Y. RGB-D sensor-based visual SLAM for localization and navigation of indoor mobile robot. In *Int. Conf. on Advanced Robotics and Mechatronics (ICARM)*, 82–87, 10.1109/ICARM.2016.7606899 (2016).

[17] Menegatti, E., Pretto, A., Scarpa, A. & Pagello, E. Omnidirectional vision scan matching for robot localization in dynamic environments. *IEEE Trans. on Robotics* **22**, 523 – 535, 10.1109/TRO.2006.875495 (2006).

[18] Bogdoll, D., Nitsche, M. & Zollner, J. M. Anomaly Detection in Autonomous Driving: A Survey. In *IEEE/CVF Conf. on Computer Vision and Pattern Recognition Workshops (CVPRW)*, 10.1109/cvprw56347.2022.00495 (IEEE, 2022).

[19] Nguyen, T.-K., Nguyen, P. T.-T., Nguyen, D.-D. & Kuo, C.-H. Effective Free-Driving Region Detection for Mobile Robots by Uncertainty Estimation Using RGB-D Data. *Sensors* **22**, 10.3390/s22134751 (2022). 10.3390/s22134751 PMC9268933 35808244

[20] Baumgartl, H. & Buettner, R. Development of a Highly Precise Place Recognition Module for Effective Human-robot Interactions in Changing Lighting and Viewpoint Conditions. In *Proc. of the 53rd Hawaii Int. Conf. on System Sciences*, 10.24251/HICSS.2020.069 (2020).

[21] Lee, M.-F. R., Hung, N. T. & Chiu, F.-H. S. An autonomous mobile robot for indoor security patrol. In *Int. Conf. on Fuzzy Theory and Its Applications (iFUZZY)*, 189–194, 10.1109/iFuzzy.2013.6825434 (2013).

[22] Espinace, P., Kollar, T., Roy, N. & Soto, A. Indoor scene recognition by a mobile robot through adaptive object detection. *Robotics and Autonomous Systems* **61**, 932–947, 10.1016/j.robot.2013.05.002 (2013).

[23] Sahdev, R. & Tsotsos, J. K. Indoor Place Recognition System for Localization of Mobile Robots. In *13th Conf. on Computer and Robot Vision (CRV)*, 53–60, 10.1109/CRV.2016.38 (2016).

[24] Alotaibi, A. *et al*. Deep Learning-Based Vision Systems for Robot Semantic Navigation: An Experimental Study. *Technologies* **12**, 157, 10.3390/technologies12090157 (2024).

[25] Zhang, X., Wang, L. & Su, Y. Visual place recognition: A survey from deep learning perspective. *Pattern Recognition* **113**, 107760, 10.1016/j.patcog.2020.107760 (2021).

[26] Lowe, D. G. Distinctive image features from scale-invariant keypoints. *Int. Journal of Computer Vision* **60**, 91–110, 10.1023/B:VISI.0000029664.99615.94 (2004).

[27] Bay, H., Tuytelaars, T. & Van Gool, L. SURF: Speeded Up Robust Features. *Computer Vision – ECCV 2006*, 404–417, 10.1007/11744023_32 (Springer, 2006).

[28] Dalal, N. & Triggs, B. Histograms of oriented gradients for human detection. In *IEEE Comp. Society Conf. on Computer Vision and Pattern Recognition (CVPR)*, vol. 1, 886–893 vol. 1, 10.1109/CVPR.2005.177 (2005).

[29] Jin, L., Gao, S., Li, Z. & Tang, J. Hand-Crafted Features or Machine Learnt Features? Together They Improve RGB-D Object Recognition. In *IEEE Int. Symposium on Multimedia, ISM 2014*, 311–319, 10.1109/ISM.2014.56 (2015).

[30] Krizhevsky, A., Sutskever, I. & Hinton, G. ImageNet classification with deep convolutional neural networks. *Neural Information Processing Systems* **25**, 10.1145/3065386 (2012).

[31] He, K., Zhang, X., Ren, S. & Sun, J. Deep Residual Learning for Image Recognition. In *IEEE Conf. on Computer Vision and Pattern Recognition (CVPR)*, 770–778, 10.1109/CVPR.2016.90 (2015).

[32] Simonyan, K. & Zisserman, A. Very deep convolutional networks for large-scale image recognition. In *3rd Int. Conf. on Learning Representations (ICLR 2015)*, 1–14 (Computational and Biological Learning Society, 2015).

[33] Radenović, F., Tolias, G. & Chum, O. Fine-tuning CNN Image Retrieval with No Human Annotation. *IEEE Trans. on Pattern Analysis and Machine Intelligence* **41**, 1655–1668, 10.1109/TPAMI.2018.2846566 (2017). 10.1109/TPAMI.2018.2846566 29994246

[34] Kolhatkar, C. & Wagle, K. Review of SLAM Algorithms for Indoor Mobile Robot with LIDAR and RGB-D Camera Technology. *Innovations in Electrical and Electronic Engineering*, 397–409, 10.1007/978-981-15-4692-1_30 (Springer, 2021).

[35] Abaspur Kazerouni, I., Fitzgerald, L., Dooly, G. & Toal, D. A survey of state-of-the-art on visual SLAM. *Expert Systems with Applications* **205**, 117734, 10.1016/j.eswa.2022.117734 (2022).

[36] Xie, T., Wang, K., Li, R. & Tang, X. Visual Robot Relocalization Based on Multi-Task CNN and Image-Similarity Strategy. *Sensors* **20**, 10.3390/s20236943 (2020). 10.3390/s20236943 PMC7730972 33291774

[37] Grimmett, H., Triebel, R., Paul, R. & Posner, I. Introspective classification for robot perception. *The Int. Journal of Robotics Research* **35**, 10.1177/0278364915587924 (2015).

[38] Pronobis, A. & Caputo, B. Confidence-based cue integration for visual place recognition. In *IEEE/RSJ Int. Conf. on Intelligent Robots and Systems*, 2394–2401, 10.1109/IROS.2007.4399493 (2007).

[39] Chandola, V., Banerjee, A. & Kumar, V. Anomaly Detection: A Survey. *ACM Comput. Surv*. **41**, 10.1145/1541880.1541882 (2009).

[40] Savage, D., Zhang, X., Yu, X., Chou, P. & Wang, Q. Anomaly detection in online social networks. *Social Networks* **39**, 62–70, 10.1016/j.socnet.2014.05.002 (2014).

[41] Laorden, C. *et al*. Study on the effectiveness of anomaly detection for spam filtering. *Information Sciences* **277**, 421–444, 10.1016/j.ins.2014.02.114 (2014).

[42] Bergmann, P., Fauser, M., Sattlegger, D. & Steger, C. MVTec AD — A Comprehensive Real-World Dataset for Unsupervised Anomaly Detection. In *IEEE/CVF Conf. on Computer Vision and Pattern Recognition (CVPR)*, 9584–9592, 10.1109/CVPR.2019.00982 (2019).

[43] Li, W., Mahadevan, V. & Vasconcelos, N. Anomaly Detection and Localization in Crowded Scenes. *IEEE Trans. on Pattern Analysis and Machine Intelligence* **36**, 18–32, 10.1109/TPAMI.2013.111 (2014). 10.1109/TPAMI.2013.111 24231863

[44] Burgard, W., Moors, M. & Schneider, F. Collaborative Exploration of Unknown Environments with Teams of Mobile Robots. *Advances in Plan-Based Control of Robotic Agents*, 52–70, 10.1007/3-540-37724-7_4 (Springer, 2002).

[45] Wu, M. & Sun, J.-Y. Moving Object Detecting and Tracking with Mobile Robot Based on Extended Kalman Filter in Unknown Environment. In *Int. Conf. on Machine Vision and Human-machine Interface*, 64–67, 10.1109/MVHI.2010.88 (2010).

[46] Henrich, D. & Gecks, T. Multi-camera collision detection between known and unknown objects. In *Sec. ACM/IEEE Int. Conf. on Distributed Smart Cameras*, 1–10, 10.1109/ICDSC.2008.4635717 (2008).

[47] Yang, J., Xu, R., Qi, Z. & Shi, Y. Visual Anomaly Detection for Images: A Systematic Survey. *Procedia Computer Science* **199**, 471–478, 10.1016/j.procs.2022.01.057 (2022).

[48] Li, J., Xu, X., Gao, L., Wang, Z. & Shao, J. Cognitive visual anomaly detection with constrained latent representations for industrial inspection robot. *Applied Soft Computing* **95**, 106539, 10.1016/j.asoc.2020.106539 (2020).

[49] Biase, G. D., Blum, H., Siegwart, R. & Cadena, C. Pixel-wise anomaly detection in complex driving scenes. In *IEEE/CVF Conf. on Computer Vision and Pattern Recognition (CVPR)*, 16913–16922, 10.1109/CVPR46437.2021.01664 (IEEE Computer Society, 2021).

[50] Nachman, B. & Shih, D. Anomaly detection with density estimation. *Phys. Rev. D* **101**, 075042, 10.1103/PhysRevD.101.075042 (2020).

[51] Khan, S. S. & Madden, M. G. One-class classification: taxonomy of study and review of techniques. *The Knowledge Engineering Review* **29**, 345–374, 10.1017/S026988891300043X (2014).

[52] Zavrtanik, V., Kristan, M. & Skočaj, D. Reconstruction by inpainting for visual anomaly detection. *Pattern Recognition* **112**, 107706, 10.1016/j.patcog.2020.107706 (2021).

[53] Li, C., Sohn, K., Yoon, J. & Pfister, T. Cutpaste: Self-supervised learning for anomaly detection and localization. In *IEEE/CVF Conf. on Computer Vision and Pattern Recognition (CVPR)*, 9659–9669, 10.1109/CVPR46437.2021.00954 (IEEE Computer Society, 2021).

[54] Japkowicz, N., Myers, C. & Gluck, M. A novelty detection approach to classification. In *Proc. of the 14th Int. Joint Conf. on Artificial Intelligence - Volume 1*, IJCAI’95, 518-523 (Morgan Kaufmann Publishers Inc., 1995).

[55] Beggel, L., Pfeiffer, M. & Bischl, B. Robust Anomaly Detection in Images Using Adversarial Autoencoders. *Machine Learning and Knowledge Discovery in Databases*, 206–222, 10.1007/978-3-030-46150-8_13 (Springer, 2020).

[56] Perera, P. & Patel, V. Learning Deep Features for One-Class Classification. *IEEE Trans. on Image Processing*, 10.1109/TIP.2019.2917862 (2018). 10.1109/TIP.2019.2917862 31144635

[57] Erfani, S., Rajasegarar, S., Karunasekera, S. & Leckie, C. High-Dimensional and Large-Scale Anomaly Detection using a Linear One-Class SVM with Deep Learning. *Pattern Recognition* **58**, 10.1016/j.patcog.2016.03.028 (2016).

[58] Schölkopf, B., Platt, J., Shawe-Taylor, J., Smola, A. & Williamson, R. Estimating Support of a High-Dimensional Distribution. *Neural Computation* **13**, 1443–1471, 10.1162/089976601750264965 (2001). 11440593 10.1162/089976601750264965

[59] Priyanga Dilini Talagala, R. J. H. & Smith-Miles, K. Anomaly Detection in High-Dimensional Data. *Journal of Computational and Graphical Statistics* **30**, 360–374, 10.1080/10618600.2020.1807997 (2021).

[60] Ismagilov, T. *et al*. On Motion Blur and Deblurring in Visual Place Recognition. *IEEE Robotics and Automation Letters* **10**, 4746–4753, 10.1109/LRA.2025.3554103 (2025).

[61] Chen, B. X. *et al*. Scene classification in indoor environments for robots using context based word embeddings. In *IEEE Int. Conf. on Robotics and Automation (ICRA) Workshop* (2018).

[62] Wozniak, P., Afrisal, H., Esparza, R. G. & Kwolek, B. Scene Recognition for Indoor Localization of Mobile Robots Using Deep CNN. *Computer Vision and Graphics*, 137–147, 10.1007/978-3-030-00692-1_13 (Springer, 2018).

[63] Wu, J., Christensen, H. I. & Rehg, J. M. Visual Place Categorization: Problem, dataset, and algorithm. In *IEEE/RSJ Int. Conf. on Intelligent Robots and Systems*, 4763–4770, 10.1109/IROS.2009.5354164 (2009).

[64] Quattoni, A. & Torralba, A. Recognizing indoor scenes. In *IEEE Conf. on Computer Vision and Pattern Recognition*, 413–420, 10.1109/CVPR.2009.5206537 (2009).

[65] Zhou, B., Lapedriza, A., Khosla, A., Oliva, A. & Torralba, A. Places: A 10 million image database for scene recognition. *IEEE Transactions on Pattern Analysis and Machine Intelligence* **40**, 1452–1464, 10.1109/TPAMI.2017.2723009 (2018). 28692961 10.1109/TPAMI.2017.2723009

[66] Pronobis, A. & Caputo, B. COLD: CoSy Localization Database. *The Int. Journal of Robotics Research (IJRR)* **28**, 588–594, 10.1177/0278364909103912 (2009).

[67] Mantegazza, D., Giusti, A., Gambardella, L. M. & Guzzi, J. An Outlier Exposure Approach to Improve Visual Anomaly Detection Performance for Mobile Robots. *IEEE Robotics and Automation Letters* **7**, 11354–11361, 10.1109/LRA.2022.3192794 (2022).

[68] Bieshaar, M., Zernetsch, S., Hubert, A., Sick, B. & Doll, K. Cooperative Starting Movement Detection of Cyclists Using Convolutional Neural Networks and a Boosted Stacking Ensemble. *IEEE Trans. on Intelligent Vehicles* **3**, 534–544, 10.1109/TIV.2018.2873900 (2018).

[69] Yuan, J. *et al*. A Novel Approach to Image-Sequence-Based Mobile Robot Place Recognition. *IEEE Trans. on Systems, Man, and Cybernetics: Systems* **51**, 5377–5391, 10.1109/TSMC.2019.2956321 (2021).

[70] Hozo, S. P., Djulbegovic, B. & Hozo, I. Estimating the mean and variance from the median, range, and the size of a sample. *BMC Medical Research Methodology* **5**, 13, 10.1186/1471-2288-5-13 (2005). 15840177 10.1186/1471-2288-5-13 PMC1097734

[71] Wozniak, P. & Ozog, D. Cross-Domain Indoor Visual Place Recognition for Mobile Robot via Generalization Using Style Augmentation. *Sensors* **23**(13), 6134, 10.3390/s23136134 (2023). 10.3390/s23136134 PMC10346347 37447982

[72] Deng, J. *et al*. ImageNet: A large-scale hierarchical image database. In *IEEE Conf. on Computer Vision and Pattern Recognition*, 248–255, 10.1109/CVPR.2009.5206848 (2009).

[73] Liu, F. T., Ting, K. M. & Zhou, Z.-H. Isolation forest. In *8th IEEE Int. Conf. on Data Mining*, 413–422, 10.1109/ICDM.2008.17 (2008).

[74] Liu, F. T., Ting, K. M. & Zhou, Z.-H. Isolation-Based Anomaly Detection. *ACM Trans. Knowl. Discov. Data* **6**, 10.1145/2133360.2133363 (2012).

[75] Cheng, Z., Zou, C. & Dong, J. Outlier Detection Using Isolation Forest and Local Outlier Factor. In *Proc. of the Conf. on Research in Adaptive and Convergent Systems*, RACS ’19, 161-168, 10.1145/3338840.3355641 (Association for Computing Machinery, 2019).

[76] Shin, H. J., Eom, D.-H. & Kim, S.-S. One-class support vector machines–an application in machine fault detection and classification. *Computers & Industrial Engineering* **48**, 395–408, 10.1016/j.cie.2005.01.009 (2005).

[77] Mũnoz-Marí, J., Bovolo, F., Gómez-Chova, L., Bruzzone, L. & Camp-Valls, G. Semisupervised One-Class Support Vector Machines for Classification of Remote Sensing Data. *IEEE Trans. on Geoscience and Remote Sensing* **48**, 3188–3197, 10.1109/TGRS.2010.2045764 (2010).

[78] Huang, C. *et al*. Self-Supervision-Augmented Deep Autoencoder for Unsupervised Visual Anomaly Detection. *IEEE Trans. on Cybernetics* **52**, 13834–13847, 10.1109/TCYB.2021.3127716 (2022). 10.1109/TCYB.2021.3127716 34851847

[79] Puggini, L. & McLoone, S. An enhanced variable selection and Isolation Forest based methodology for anomaly detection with OES data. *Engineering Applications of Artificial Intelligence* **67**, 126–135, 10.1016/j.engappai.2017.09.021 (2018).

[80] Xu, H., Pang, G., Wang, Y. & Wang, Y. Deep Isolation Forest for Anomaly Detection. *IEEE Trans. on Knowledge and Data Engineering* **35**, 12591–12604, 10.1109/TKDE.2023.3270293 (2023).

[81] Luan, S., Gu, Z., Freidovich, L. B., Jiang, L. & Zhao, Q. Out-of-Distribution Detection for Deep Neural Networks With Isolation Forest and Local Outlier Factor. *IEEE Access* **9**, 132980–132989, 10.1109/ACCESS.2021.3108451 (2021).

[82] Yin, S., Zhu, X. & Jing, C. Fault detection based on a robust one class support vector machine. *Neurocomputing* **145**, 263–268, 10.1016/j.neucom.2014.05.035 (2014).

[83] Tax, D. & Duin, R. Support Vector Data Description. *Machine Learning* **54**, 45–66, 10.1023/B:MACH.0000008084.60811.49 (2004).

[84] Perera, P. & Patel, V. M. Deep Transfer Learning for Multiple Class Novelty Detection. In *IEEE/CVF Conf. on Computer Vision and Pattern Recognition (CVPR)*, 11536–11544, 10.1109/CVPR.2019.01181 (2019).

[85] Oza, P. & Patel, V. One-Class Convolutional Neural Network. *IEEE Signal Processing Letters* **26**, 277–281, 10.1109/LSP.2018.2889273 (2018).

[86] Wang, J., He, H. & Prokhorov, D. V. A Folded Neural Network Autoencoder for Dimensionality Reduction. *Procedia Computer Science* **13**, 120–127, 10.1016/j.procs.2012.09.120*Proc. of the Int. Neural Network Society Winter Conf. (INNS-WC2012)* (2012).

[87] Meng, Q., Catchpoole, D., Skillicom, D. & Kennedy, P. J. Relational autoencoder for feature extraction. In *Int. Joint Conf. on Neural Networks (IJCNN)*, 364–371, 10.1109/IJCNN.2017.7965877 (2017).

[88] Zhou, L., Cai, C., Gao, Y., Su, S. & Wu, J. Variational autoencoder for low bit-rate image compression. In *Proc. of the IEEE Conf. on Computer Vision and Pattern Recognition Workshops*, 2617–2620 (2018).

[89] Kavitha, K., Sandhya, B. & Thirumala, B. Evaluation of Distance Measures for Feature based Image Registration using AlexNet. *Int. Journal of Advanced Computer Science and Applications* **9**, 10.14569/ijacsa.2018.091034 (2018).

[90] Wozniak, P., Krzeszowski, T. & Kwolek, B. Multi-Domain Dataset for Robots (MDDRobots) - Multi-Domain Indoor Dataset for Visual Place Recognition and Anomaly Detection by Mobile Robots, 10.5281/zenodo.11504581 (2025). 10.1038/s41597-025-05124-3 PMC12089276 40389441

[91] Ruiz-Sarmiento, J., Galindo, C. & González-Jiménez, J. Robot@Home, a robotic dataset for semantic mapping of home environments. *The Int. Journal of Robotics Research* **36**, 027836491769564, 10.1177/0278364917695640 (2017).

[92] Mantegazza, D., Xhyra, A., Gambardella, L. M., Giusti, A. & Guzzi, J. Hazards&Robots: A dataset for visual anomaly detection in robotics. *Data in Brief* **48**, 109264, 10.1016/j.dib.2023.109264 (2023). 37383812 10.1016/j.dib.2023.109264 PMC10294035

